# Urinary albumin excretion in healthy adults: a cross sectional study of 24-hour versus timed overnight samples and impact of GFR and other personal characteristics

**DOI:** 10.1186/1471-2369-16-8

**Published:** 2015-01-24

**Authors:** Peter Fagerstrom, Gerd Sallsten, Magnus Akerstrom, Borje Haraldsson, Lars Barregard

**Affiliations:** Department of Occupational and Environmental Medicine, Sahlgrenska Academy, University of Gothenburg, PO Box 414, Gothenburg, S-405 30 Sweden; Department of Molecular and Clinical Medicine, Sahlgrenska Academy, University of Gothenburg, Gothenburg, Sweden

**Keywords:** Glomerular filtration rate, Urinary albumin, Timed urine samples, 24-hour samples, Overnight samples

## Abstract

**Background:**

Urinary albumin can be measured in 24 h or spot samples. The 24 h urinary albumin excretion rate is considered the gold standard, but is cumbersome to collect. Instead, often an overnight sample is collected, and adjusted for dilution. Proxies for 24 h excretion rate have been studied in diabetics, but seldom in healthy individuals. Our aims were to compare 24 h and overnight albumin excretion, to assess the impact of personal characteristics, and to examine correlations between the 24 h excretion rate and proxies such as the albumin to creatinine ratio (ACR).

**Methods:**

Separate 24 h and overnight urine samples were collected from 152 healthy kidney donors. Urinary creatinine, specific gravity, collection time, and sample volume determined. Differences between 24 h and overnight samples were examined, and the effects of age, sex, smoking, body mass, glomerular filtration rate, and urinary flow rate were assessed.

**Results:**

The 24 h albumin excretion rate and ACR were both significantly higher than their overnight counterparts. Unadjusted albumin was unsurprisingly higher in the more concentrated overnight samples, while concentrations adjusted for specific gravity were similar. In multivariate analysis, the 24 h excretion rate and proxies were positively associated with glomerular filtration rate, as was ACR in overnight samples. There were positive associations between urinary albumin and body mass.

**Conclusions:**

Proxies for the 24 h albumin excretion rate showed relatively high correlations with this gold standard, but differences due to sampling period, adjustment method, and personal characteristics were large enough to be worth considering in studies of albumin excretion in healthy individuals.

## Background

The detection and quantification of albumin in urine is common and important in clinical medical practice. It is widely used for screening of diabetic and hypertensive nephropathy, as well as preeclampsia. Microalbuminuria is also a risk factor for cardiovascular morbidity and mortality [1–5].

The urine sample used to measure albumin can be collected during a 24 h period, overnight (ON), or as a spot urine sample at any time of the day. It is still unclear how albumin excretion differs between 24 h and ON samples in a healthy population. The 24 h albumin excretion rate (AER, mg/h) is considered to be the gold standard, although the need for timed samples is still debated [6–8]. The simpler and therefore more widely used urinary albumin concentration (UAC, mg/L) can be adjusted for dilution, either using urinary creatinine (albumin to creatinine ratio, ACR, mg/g) or urine specific gravity (SG, albumin adjusted for specific gravity, ASG, mg/L).

There are many studies on urinary albumin (U-Alb) in diabetes and various other clinical conditions, but there is a notable lack of studies examining different ways of expressing albumin excretion in healthy individuals [9, 10]. Such information is important, for example, when urinary albumin is used in epidemiological studies searching for mild effects on renal function of nephrotoxic drugs or environmental contaminants, and in studies assessing associations between U-Alb and risk of cardiovascular disease.

This study had three aims:

1. 24 h sampling vs. overnight sampling: To examine associations and differences between U-Alb in 24 h and ON samples measured as AER, UAC, ACR, and ASG.
2. Determinants in healthy subjects: To investigate how glomerular filtration rate (GFR), sex, age, body mass, smoking, and urinary flow (UF) affect U-Alb measured as AER, UAC, ACR, and ASG.
3. Proxies: To investigate how well UAC, ACR, and ASG in ON samples reflect 24 h AER.

## Methods

### Study population

Between 1999 and 2005, 152 healthy kidney donors (no kidney disease or diabetes, and normal GFR) were recruited and examined as previously described [11, 12]. Background data on these individuals are shown in Table 1. Informed consent was obtained from the participants. The study adhered to the Declaration of Helsinki and was approved by the ethical review board of the University of Gothenburg.

**Table 1:** **Background data on participants and urine samples (means and ranges)**

	All	Men	Women
N	152	65	87
Never-/ever-smokers (n)	60/91	27/38	33/53
Pack-years^a^	13.8 (0.4-51.0)	14.5 (0.4-51.0)	13.3 (1.2-36.3)
Body mass (lbs)	165 (108–245)	183 (143–245)	150 (108–209)
Age	48 (24–70)	49 (30–70)	48 (24–64)
GFR (ml/min/1.73 m^2^)	101 (77.0-147)	99.5 (78-140)	101 (77.0-147)
24 h samples (n)	130	55	75
24 h collection time (hrs)	23.2 (20.5-26.6)	23.3 (21.5-26.6)	23.1 (20.5-24.8)
24 h volume (ml)	1878 (444–5790)	1795 (680–4199)	1938 (444–5790)
ON samples (n)	145	63	82
ON collection time (hrs)	8.7 (6.0-14.8)	8.7 (6.0-14.8)	8.6 (6.3-11.3)
ON volume (ml)	419 (95–1050)	434 (120-1050)	407 (95–1002)

^a^in ever-smokers.

### Urine samples and biochemical assays

As previously described, two separate timed urine samples were collected from each of the participants on two consecutive days, one 24 h and one ON sample [11, 12]. In this collection, 123 participants provided both ON and 24 h samples, 22 provided only an ON sample, and 7 provided only a 24 h sample. Time since last urination (collection time) and sample volume were recorded (Table 1).

Urinary albumin and creatinine concentrations were determined at the Department of Clinical Chemistry, Sahlgrenska University hospital, Gothenburg. Urinary albumin concentrations were determined on fresh samples by an automated nephelometric immunochemical method using reagents and calibrator from Beckman Coulter (Fullerton, CA, USA). Internal reference samples were used in each analytical run. The detection limit was 2.4 mg/L. Urinary creatinine concentrations were determined using the Jaffé method (Roche Diagnostics, GmbH, Mannheim, Germany) in the first three batches, and an enzymatic method (Modular P and CREAplus R1, R2, Roche/Hitachi, Roche Diagnostics, GmbH, Mannheim, Germany) in the last batch (N = 41). The methods are comparable [13]. In addition, the laboratory performed internal quality control measures when introducing the enzymatic method, in order to ascertain that results were comparable. Specific gravity was measured with a refractometer (Medline, Ceti, Digit 012, Oxfordshire, UK).

GFR was measured using plasma clearance of iohexol or Cr-EDTA, except for a few cases where technetium-99 m-DTPA was used. The methods are comparable as GFR markers [14].

AER, ACR, ASG, and UF were calculated from the UAC, sample volume, collection time, specific gravity, and creatinine concentration. ASG was adjusted to SG = 1.015 according to the formula ASG = U-Alb*(1.015-1)/(specific gravity-1) [15].

### Statistics

UAC values below the detection limit (23 ON samples and 41 24 h samples) were replaced by detection limit/√2 = 2.4 /√2 = 1.7 [16]. Statistical analyses were performed using version 9.2 of the SAS software package (SAS Institute, Cary, NC, USA). Several variables were not normally distributed. Associations between variables were tested with the Spearman correlation coefficient and with linear and multiple linear regression analysis. Differences between 24 h and ON samples were tested using paired t-tests. Differences between groups were tested using the Wilcoxon rank sum test. Statistical significance was determined at P<0.05 (two-tailed). All multiple regression analyses were performed stepwise as shown below.
1

## Results

### Albumin excretion rate (AER)

**Figure 1:**
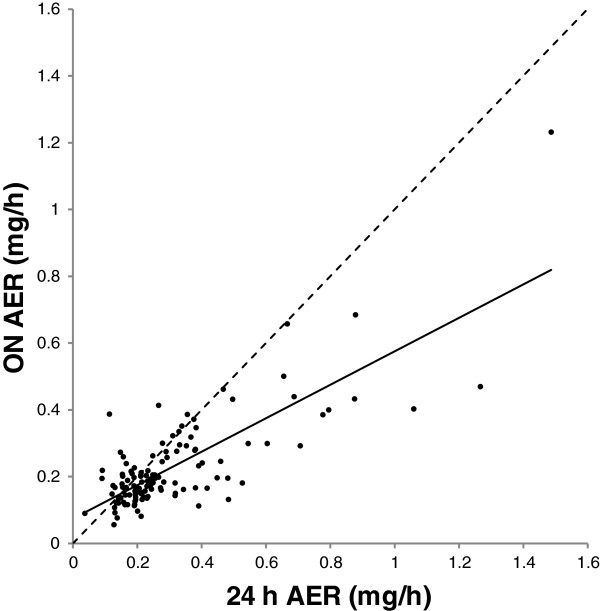
**Association between albumin excretion rate in overnight and 24 h urine.** Association between albumin excretion rate (AER) in overnight (ON) and 24 h urine, both in mg/h. Regression line is shown: ON AER=0.075+0.50*24 h AER. R^2^=0.62, P<0.001. Hatched line: line of identity. *Note:* Conversion factor for AER in mg/h to mg/d, *24.

The 24 h albumin excretion rate was significantly *higher* than the ON AER (Table 2). The association between 24 h and ON AER is shown in Figure 1.

**Table 2:** **Excretion rates and concentrations of albumin in 24 h and overnight (ON) samples**

	N	Mean	Range	P-value difference 24 h/overnight
Albumin excretion				
24 h AER (mg/24 h)	130	7.46	0.88-35.7	<0.001
ON AER (mg/24 h)	145	5.85	0.81-29.6	
Albumin concentration				
24 h UAC (mg/L)	130	4.30	1.70-22.0	<0.001
ON UAC (mg/L)	145	6.33	1.70-45.0	
24 h ACR (mg/g Cr)	130	5.85	1.36-24.3	<0.001
ON ACR (mg/g Cr)	145	4.94	0.83-24.9	
24 h ASG (mg/L)	130	4.26	0.67-20.0	0.93
ON ASG (mg/L)	145	4.46	0.98-27.3	
Creatinine concentration				
24 h UCC (g/L)	130	0.77	0.18-3.94	<0.001
ON UCC (g/L)	145	1.28	0.33-2.49	
Specific gravity				
24 h SG	130	1.016	0.005-1.038	<0.001
ON SG	145	1.021	1.006-1.040	
Urinary flow rate				
24 h UF (mL/h)	130	81.2	20.4-246	<0.001
ON UF (mL/h)	145	49.2	11.5-150	
Creatinine excretion rate				
24 h CER (mg/24 h)	130	11.6	4.69-21.8	<0.001
Overnight CER (mg/24 h)	145	11.0	3.07-19.6	

*Note:* Differences between 24 h and ON samples were calculated for 123 paired samples. *Abbreviations:* *AER* Albumin excretion rate, *UAC* Urinary albumin concentration, *ACR* Albumin to creatinine ratio, *Cr* Creatinine, *ASG* Albumin adjusted for specific gravity, *UCC* Urinary creatinine concentration, *SG* Specific gravity, *UF* Urinary flow.

There was a positive association between GFR and 24 h AER, but not between GFR and ON AER (Table 3). There was a positive correlation between body mass and AER in both 24 h and ON samples. There was no significant correlation between UF and AER. Men had higher ON AER than women, but no significant difference was seen in 24 h AER (Table 4). No difference was seen between never-smokers and ever-smokers.

**Table 3:** **Spearman correlation coefficients between 24 h AER, proxies and determinants**

	24 h AER	24 h UAC	24 h ACR	24 h ASG	ON AER	ON UAC	ON ACR	ON ASG
24 h AER	1.00	0.77	0.77	0.71	0.61	0.40^a^	0.42^b^	0.40
		<0.001	<0.001	<0.001	<0.001	<0.001	<0.001	<0.001
	130	130	130	130	123	123	123	123
24 h UAC	0.77	1.00	0.63	0.69	0.57	0.60	0.42	0.48
	<0.001		<0.001	<0.001	<0.001	<0.001	<0.001	<0.001
	130	130	130	130	123	123	123	123
24 h ACR	0.77	0.63	1.00	0.75	0.44	0.31	0.67	0.41
	<0.001	<0.001		<0.001	<0.001	.0005	<0.001	<0.001
	130	130	130	130	123	123	123	123
24 h ASG	0.71	0.69	0.75	1.00	0.50	0.40	0.54	0.59
	<0.001	<0.001	<0.001		<0.001	0.005	<0.001	<0.001
	130	130	130	130	123	123	123	123
GFR	0.24	0.21	0.18	0.08	0.05	0.07	0.04	0.06
	0.006	0.02	0.04	0.35	0.58	0.39	0.67	0.49
	130	130	130	130	144	144	144	144
Sex	-0.12	-0.17	0.33	0.07	-0.28	-0.09	0.21	0.08
	0.16	0.06	<0.001	0.40	0.007	0.26	0.01	0.92
	130	130	130	130	145	145	145	145
Body mass	0.17	0.21	-0.22	0.03	0.30	0.11	-0.14	0.09
	0.04	0.02	0.01	0.79	<0.001	0.21	0.05	0.34
	128	128	128	128	142	142	142	142
Smoking	-0.04	-0.09	0.01	-0.15	-0.03	-0.08	0.05	-0.02
	0.61	0.33	0.88	0.09	0.72	0.34	0.52	0.83
	129	129	129	129	144	144	144	144
Age	-0.08	0.06	0.06	-0.02	0.13	0.09	0.23	0.09
	0.38	0.50	0.53	0.83	0.12	0.30	0.006	0.27
	130	130	130	130	145	145	145	145
UF	0.06^c^	-0.51^c^	-0.006^c^	-0.11^c^	-0.03^d^	-0.70^d^	-0.18^d^	-0.41^d^
	0.47	<0.001	0.94	0.21	0.68	<0.001	0.04	<0.001
	130	130	130	130	145	145	145	145
UCC	0.1^e^	0.54^e^	-0.24^e^	0.05^e^	0.24^f^	0.71^f^	-0.06^f^	0.35^f^
	0.10	<0.001	0.005	0.56	0.04	<0.001	0.94	<0.001
	130	130	130	130	145	145	145	145

*Note:* First row in each cell, spearman correlation coefficient; second row, P-value; third row, number of observations.

*Abbreviations:* *ON* Overnight, *AER* Albumin excretion rate, *UAC* Urinary albumin concentration, *ASG* Albumin adjusted for specific gravity, *ACR* Albumin to creatinine ratio, *GFR* Glomerular filtration rate, *UF* Urinary flow, *UCC* Urinary creatinine concentration.

^a^Men: r=0.59, P<0.001, N=53; Women: r=0.24, P=0.043, N=70. ^b^Men: r=0.64, P<0.001, N=53; Women: r=0.37, P=0.002, N=70. ^c^24 h UF. ^d^ON UF. ^e^24 h UCC. ^f^ON UCC.

**Table 4:** **Differences between men and women and between ever-smokers and never-smokers**

	Women	Men		Ever-smokers	Never-smokers	
	Mean	Mean	P^a^	Mean	Mean	P^a^
24 h AER (mg/24 h)	6.95	8.16	0.16	7.05	7.99	0.61
ON AER (mg/24 h)	5.59	6.19	<0.001	5.47	6.50	0.71
24 h UAC (mg/L)	3.93	4.80	0.06	3.91	4.90	0.33
ON UAC (mg/L)	6.51	6.10	0.26	5.69	7.42	0.34
24 h ACR (mg/g Cr)	6.37	5.13	0.001	5.81	5.84	0.09
ON ACR (mg/g Cr)	5.55	4.15	0.004	4.73	5.29	0.52
24 H ASG mg/L)	4.33	4.15	0.40	4.00	4.66	0.16
ON ASG (mg/L)	4.81	4.01	0.92	4.18	4.96	0.83
24 h UCC (g/L)	0.61	0.99	<0.001	1.20	0.87	0.03
ON UCC (g/L)	1.12	1.49	<0.001	1.02	1.42	0.04
24 h SG	1.014	1.018	<0.001	1.016	1.016	0.96
ON SG	1.019	1.023	0.008	1.020	1.022	0.12

*Abbreviations:* *ON* Overnight, *AER* Albumin excretion rate, *UAC* Urinary albumin concentration, *ASG* Albumin adjusted for specific gravity, *ACR* Albumin to creatinine ratio, *Cr* Creatinine, *GFR* Glomerular filtration rate, *UCC* Urinary creatinine concentration, *SG* Specific gravity.

^a^Wilcoxon rank sum test.

In a multiple regression analysis according to Equation 1 the positive association between GFR and the 24 h AER remained (P = 0.003) but there was no significant association with ON AER (P = 0.09). Body mass was associated with ON AER (P = 0.02), but not with 24 h AER (P = 0.06).

### Urinary albumin concentration (UAC)

**Figure 2:**
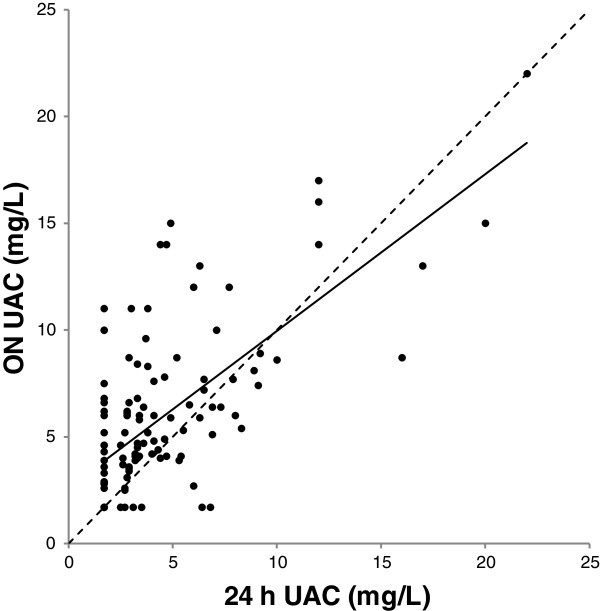
**Association between urinary albumin concentration in overnight and 24 h urine.** Association between urinary albumin concentration (UAC) in overnight (ON) and 24 h urine, both in mg/L. Regression line is shown: ON UAC=0.73+0.62*24 h UAC. R^2^=0.46, P<0.001. Hatched line: line of identity. *Note:* Conversion factor for UAC in mg/L to g/dL, *10^-4^.

**Figure 3:**
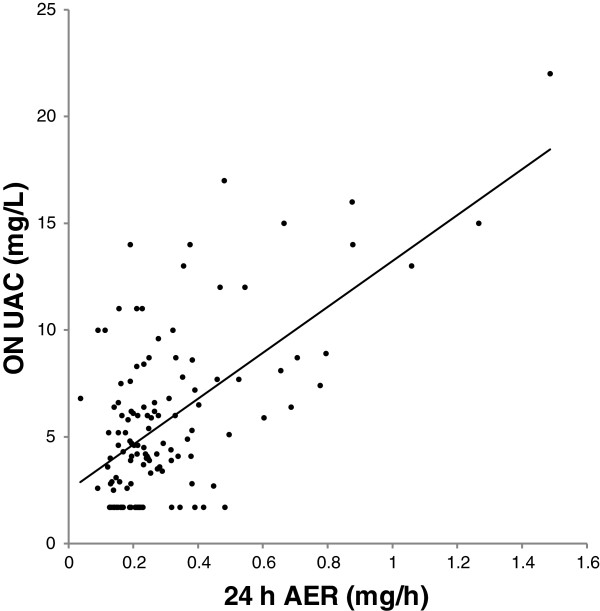
**Association between overnight urinary albumin concentration and 24 h albumin excretion rate.** Association between overnight urinary albumin concentration (ON UAC) and 24 h albumin excretion rate (24 h AER). Regression line is shown: ON UAC=2.47+10.8*24 h AER. R^2^=0.38, P<0.001. *Note:* Conversion factor for UAC in mg/L to g/dL, *10^-4^. Conversion factor for AER in mg/h to mg/d, *24.

As expected, the 24 h UAC was significantly *lower* than UAC in the more concentrated ON samples (Table 2). The association between ON and 24 h UAC is shown in Figure 2 and the association between ON UAC and 24 h AER in Figure 3.

Again as expected, both ON and 24 h UAC showed significant negative correlations with UF (Table 3). The 24 h UAC was positively associated with GFR and body mass, while this was not the case for ON UAC. Men tended to have higher 24 h UAC than women (P = 0.06), but no difference was seen in ON UAC (Table 4). No difference was seen between never-smokers and ever-smokers.

A multiple regression analysis according to Equation 1 showed a significant negative association between UAC and UF in 24 h (P = 0.002) and ON (P<0.001) samples. There were also positive associations with GFR (P = 0.005) for 24 h UAC, but not significantly so for ON UAC (P = 0.08). Ever-smokers had higher 24 h UAC (P = 0.049) than never-smokers. There was no significant impact of age, sex, or body mass.

### Albumin to creatinine concentration (ACR)

**Figure 4:**
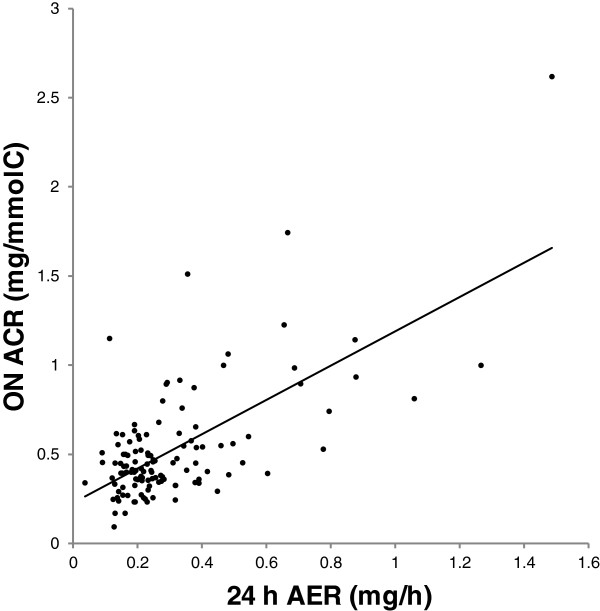
**Association between overnight urinary albumin to creatinine ratio and 24 h albumin excretion rate.** Association between overnight urinary albumin to creatinine ratio (ON ACR) and 24 h albumin excretion rate (24 h AER). Regression line is shown: ON ACR=0.23+0.96*24 h AER. R^2^=0.44, P<0.001. *Note:* Conversion factor for ACR in mg/mmol to mg/g, *8.84. Conversion factor for AER in mg/h to mg/d, *24.

The 24 h ACR was significantly higher than ON ACR (Table 2; regression equation: ON ACR = 0.15+0.58*24). The association between ON ACR and 24 h AER is shown in Figure 4. The 24 h ACR but not the ON ACR was negatively correlated with the urinary creatinine concentration (Table 3). The 24 h ACR was positively correlated with GFR and negatively correlated with body mass. The ON ACR was positively correlated with age and negatively correlated with body mass. Both 24 h and ON ACR were higher in women than in men (Table 4). No significant difference was seen between never-smokers and ever-smokers.

In a multiple regression analysis according to Equation 1, the associations with sex (higher in women; 24 h ACR: P = 0.03; ON ACR P = 0.02) and GFR (24 h ACR: P = 0.001; ON ACR P = 0.04) remained, while there was no significant impact of age, body mass, or smoking. ON UF was not significantly associated with ON ACR (P = 0.09).

### Albumin adjusted for specific gravity (ASG)

The 24 h and ON ASG were not significantly different (Table 2). The associations between 24 h and ON ASG on the one hand and the 24 h AER on the other were similar to those for the corresponding UAC and ACR measures (Table 3). No significant difference was seen between men and women or between never-smokers and ever-smokers (Table 4).

A multiple regression analysis according to Equation 1 showed a positive association between GFR and 24 h ASG (P = 0.03). ON ASG was negatively associated with UF (P = 0.01).

## Discussion

The present study showed that AER was higher in 24 h than in ON samples. ON samples may thus cause an underestimation (about 25%) of the true albumin excretion (Table 3 and Figure 1). To our knowledge, only two previous studies have examined diurnal variation of albumin excretion in healthy subjects, both showing lower excretion in ON samples [17, 18]. The reason for this difference may include daytime erect position (postural proteinuria) or physical exercise [18–21]. However, Montagna et al. also found a variation independent of posture and activity [18], which may be explained by diurnal variation of GFR secondary to diurnal variation in blood pressure [22, 23].

We also found that 24 h AER was significantly positively correlated with GFR, and there was a similar tendency for ON AER. This finding is in agreement with rat experiments by Ohlson et al. [24] Some authors have, however, found that the glomerular filtration of albumin is relatively independent of GFR, while the urinary excretion of albumin is more dependent on possible saturation of tubular reabsorption [25]. An explanation of the different results could be that we studied inter-individual variation in GFR while Smithies discussed intra-individual variation [25].

AER was associated with body mass, in agreement with previous studies showing an association between 24 h AER and BMI [5, 26].

We found no significant association between AER and UF. However, it has been shown that water loading does increase AER, perhaps because the reuptake mechanisms are less effective, or possibly because of increased GFR due to increased blood pressure [27]. Our findings suggest that this does not occur during physiological variations in UF.

The 24 h AER was not significantly different in women and men, while the ON AER was slightly higher in men. The explanation may be the higher body mass in men, since in the multiple regression model body mass, but not sex, had a significant impact on AER.

There was no difference in AER between never-smokers and ever-smokers, a finding in line with previous inconclusive studies [28]. We found no significant correlation between AER and age, in agreement with most previous studies [28, 29].

The ON UAC was higher than 24 h UAC, despite the fact that the AER was lower in ON than 24 h samples. The difference between ON and 24 h UAC samples must therefore have been caused by dilution (higher UF; see Table 2). UAC was significantly positively correlated with GFR in a multiple regression analysis, as was AER.

The association between ON UAC and 24 h AER was only moderate (R^2^ = 0.38, Figure 3), although the correlation was not much lower than for ON ACR and ON ASG (Table 3). This indicates that creatinine adjustment may not be necessary if ON samples are used. ON UAC has been suggested to replace ACR as screening method [30–35]. The situation is different in daytime spot samples, which may be much diluted.

The 24 h ACR was higher than ON ACR, reflecting the corresponding difference seen for AER. ACR was significantly positively correlated with GFR in a multiple regression analysis, as was AER.

Random spot ACR have been used and there is evidence that they can adequately predict 24 h urinary protein loss in diabetics and others with kidney disease [9]. However, there are no studies known to us that shows this in healthy individuals. Previous studies have shown that U-Alb varies not only between day and night, but during the day as well, and this variability must be taken into consideration when interpreting the results [9, 36]. Overnight samples have a longer collection period than day-time spot samples, and physical activity, posture and intake of fluids varies less during the night than in the day. Therefore we believe that ON samples are to prefer over random day-time samples when evaluating U-Alb. As expected, there was a significant difference in concentrations and excretion rates of creatinine (data not shown) and ACR between men and women. This is known to be caused by higher muscle mass in men [37–39]. Although ON UF was positively correlated with ON creatinine excretion rate, UF did not have any impact on ACR. ON ACR was the only albumin measure we found to correlate with age [40, 41], probably due to lower muscle mass at higher age.

ON ACR, which is widely used for screening purposes, showed a somewhat higher correlation with 24 h AER (R^2^ = 0.44) than did ON UAC. ON ACR is relatively easy to collect and measure, which makes it suitable to use for screening and follow up in individual patients [6, 42–44]. Lambers Heerspink et al. showed that first morning ACR was just as good as 24 h AER for prediction of cardiovascular disease [42], and suggested that this may be due to many errors in collection of 24 h samples. However, ACR may generate some falsely high ACR levels in patients with low muscle mass and hence low creatinine excretion [37]. ACR is a well-established measure, but has not been studied in all populations, and most of the available data are on diabetics.

There was no difference between 24 h and ON ASG, probably because ON SG was significantly higher than 24 h SG (Table 2). Thus, adjusting for SG did not capture the true difference between ON and 24 h AER.

ASG is sensitive to hematuria and glucosuria, since they affect specific gravity [45, 46]. On the other hand, we found little or no correlation between ASG and other variables in this study, such as age, sex, and body mass.

Although its routine use is rare, ASG could be used instead of ACR, especially in situations where creatinine excretion varies due to factors such as changes in body composition and protein intake [47]. However, SG may be affected by intake of certain food, like salt. Elkins et al. also found that creatinine adjustment is more suitable for very diluted or concentrated samples [46].

A strength of this study is the fact that separate ON and 24 hour samples were collected; that is, the ON sample was not included in the 24 hour sample [10]. U-Alb is known to have intra-individual variation of 44-85 % (between days), depending on sample types [40, 48–50]. Despite this variability, we could show a difference between ON and 24 h samples collected on separate days. If the ON sample had been a part of the 24 h sample, we would have had better power to quantify differences between ON and 24 h samples.

The samples were collected when the study subjects were hospitalized, which reduced the risk of contamination and collection errors. It is often recommended that very concentrated (U-Crea>3 g/L) or dilute (U-Crea<0.3 g/L) samples should be excluded, since the validity of such samples could be questioned. However, in the present study, such urine samples were included, since one of the aims was to study the impact of UF on albumin excretion.

The albumin concentrations were analyzed in fresh urine samples. It has been shown that freezing and thawing of urine can underestimate and increase the variability of measured U-Alb.

Another strength is the fact that we had data on measured GFR. To the best of our knowledge, associations between GFR and U-Alb in healthy subjects have not been reported previously. Estimated GFR (eGFR) is widely used, and there are several different equations to obtain this measure. It is however important to remember that while it is useful for population studies, eGFR is a blunt tool for use in individual patients.

A limitation is the fact that the study was performed on kidney donors, who are somewhat ‘more healthy’ that a general population sample. People with diabetes, hypertension, or kidney disease were not included in our sample. On the other hand, as shown in Table 1, our sample was diverse enough to include a person of 70 years of age and a long-term heavy smoker.

## Conclusions

The 24 h AER is the gold standard, even though other measures of U-Alb may work relatively well. ON AER will underestimate the 24-hour albumin excretion, probably due to nocturnal hypotension and subsequent reduction of glomerular filtration pressure. In addition, GFR and body mass are positively associated with AER and ACR. The differences between the various estimates of albumin excretion are large enough to be worth considering in scientific studies in healthy adults.

## References

[1] Klausen K, Borch-Johnsen K, Feldt-Rasmussen B, Jensen G, Clausen P, Scharling H (2004). Very low levels of microalbuminuria are associated with increased risk of coronary heart disease and death independently of renal function, hypertension, and diabetes. Circulation.

[2] Romundstad S, Holmen J, Kvenild K, Hallan H, Ellekjær H (2003). Microalbuminuria and all-cause mortality in 2,089 apparently healthy individuals: a 4.4-year follow-up study. The nord-trøndelag health study (HUNT), Norway. Am J Kidn Dis.

[3] Ärnlöv J, Evans JC, Meigs JB, Wang TJ, Fox CS, Levy D (2005). Low-grade albuminuria and incidence of cardiovascular disease events in nonhypertensive and nondiabetic individuals: The Framingham Heart Study. Circulation.

[4] Damsgaard EM, Frøland A, Jørgensen OD, Mogensen CE (1990). Microalbuminuria as predictor of increased mortality in elderly people. BMJ.

[5] Verhave JC, Hillege HL, Burgerhof JGM, Navis G, de Zeeuw D, de Jong PE (2003). Cardiovascular risk factors are differently associated with urinary albumin excretion in men and women. J Am Soc Nephrol.

[6] Gaspari F, Perico N, Remuzzi G (2006). Timed urine collections are not needed to measure urine protein excretion in clinical practice. Am J Kidn Dis.

[7] Shidham G, Hebert L (2006). Timed urine collections are not needed to measure urine protein excretion in clinical practice. Am J Kidn Dis.

[8] Naresh CN, Hayen A, Weening A, Craig JC, Chadban SJ (2013). Day-to-Day variability in spot urine albumin-creatinine ratio. Am J Kidney Dis.

[9] Guy M, Borzomato JK, Newall RG, Kalra PA, Price CP (2009). Protein and albumin-to-creatinine ratios in random urines accurately predict 24 h protein and albumin loss in patients with kidney disease. Ann Clin Biochem.

[10] Gansevoort RT, Brinkman J, Bakker SJL, De Jong PE, de Zeeuw D (2006). Evaluation of measures of urinary albumin excretion. Am J Epidemiol.

[11] Barregard L, Fabricius-Lagging E, Lundh T, Mölne J, Wallin M, Olausson M (2010). Cadmium, mercury, and lead in kidney cortex of living kidney donors: Impact of different exposure sources. Environ Res.

[12] Akerstrom M, Lundh T, Barregard L, Sallsten G (2012). Sampling of urinary cadmium: differences between 24-h urine and overnight spot urine sampling, and impact of adjustment for dilution. Int Arch Occup Environ Health.

[13] Junge W, Wilke B, Halabi A, Klein G (2004). Determination of reference intervals for serum creatinine, creatinine excretion and creatinine clearance with an enzymatic and a modified Jaffé method. Clin Chim Acta.

[14] Brändström E, Grzegorczyk A, Jacobsson L, Friberg P, Lindahl A, Aurell M (1998). GFR measurement with iohexol and 51Cr-EDTA. A comparison of the two favoured GFR markers in Europe. Nephrol Dial Transplant.

[15] Suwazono Y, Åkesson A, Alfvén T, Järup L, Vahter M (2005). Creatinine versus specific gravity-adjusted urinary cadmium concentrations. Biomarkers.

[16] Hornung RW, Reed LD (1990). Estimation of average concentration in the presence of nondetectable values. Appl Occup Environ Hyg.

[17] Watts GF, Morris RW, Khan K, Polak A (1988). Urinary albumin excretion in healthy adult subjects: Reference values and some factors affecting their interpretation. Clin Chim Acta.

[18] Montagna G, Buzio C, Calderini C, Quadetti P, Migone L (1983). Relationship of proteinuria and albuminuria to posture and to urine collection period. Nephron.

[19] Robinson RR, Glenn WG (1964). Fixed and reproducible orthostatic proteinuria. IV. Urinary albumin excretion by healthy human subjects in the recumbent and upright postures. J Lab Clin Med.

[20] Junglee NA, Lemmey AB, Burton M, Searell C, Jones D, Lawley JS (2012). Does proteinuria-inducing physical activity increase biomarkers of acute kidney injury?. Kidney Blood Press Res.

[21] Newman DJ, Pugia MJ, Lott JA, Wallace JF, Hiar AM (2000). Urinary protein and albumin excretion corrected by creatinine and specific gravity. Clin Chim Acta.

[22] Koopman MG, Koomen GC, Krediet RT, de Moor EA, Hoek FJ, Arisz L (1989). Circadian rhythm of glomerular filtration rate in normal individuals. Clin Sci (Lond).

[23] Yamasaki F, Schwartz JE, Gerber LM, Warren K, Pickering TG (1998). Impact of shift work and race/ethnicity on the diurnal rhythm of blood pressure and catecholamines. Hypertension.

[24] Ohlson M, Sörensson J, Lindström K, Blom AM, Fries E, Haraldsson B (2001). Effects of filtration rate on the glomerular barrier and clearance of four differently shaped molecules. Am J Physiol Renal Physiol.

[25] Smithies O (2003). Why the kidney glomerulus does not clog: A gel permeation/diffusion hypothesis of renal function. Proc Natl Acad Sci U S A.

[26] Cirillo M, Senigalliesi L, Laurenzi M (1998). Microalbuminuria in nondiabetic adults: Relation of blood pressure, body mass index, plasma cholesterol levels, and smoking: the gubbio population study. Arch Intern Med.

[27] Viberti GC, Mogensen CE, Keen H, Jacobsen FK, Jarrett RJ, Christensen CK (1982). Urinary excretion of albumin in normal man: The effect of water loading. Scand J Clin Lab Invest.

[28] Gosling P, Beevers DG (1989). Urinary albumin excretion and blood pressure in the general population. Clin Sci (Lond).

[29] Jensen JS, Feldt-rasmussen B, Borch-johnsen K, Jensen G (1993). Urinary albumin excretion in a population based sample of 1011 middle aged non-diabetic subjects. Scand J Clin Lab Invest.

[30] Hutchison AS, O'Reilly DS, MacCuish AC (1988). Albumin excretion rate, albumin concentration, and albumin/creatinine ratio compared for screening diabetics for slight albuminuria. Clin Chem.

[31] Derhaschnig U, Kittler H, Woisetschläger C, Bur A, Herkner H, Hirschl MM (2002). Microalbumin measurement alone or calculation of the albumin/creatinine ratio for the screening of hypertension patients?. Nephrol Dial Transplant.

[32] Kallner A, Estonius M (2005). Are there advantages with U-Albumin/U-Creatinine ratios compared with U-Albumin in monitoring diabetes?. Scand J Clin Lab Invest.

[33] Cowell C, Rogers S, Silink M (1986). First morning urinary albumin concentration is a good predictor of 24-hour urinary albumin excretion in children with Type I (insulin-dependent) diabetes. Diabetologia.

[34] Howey JE, Browning MC, Fraser CG (1987). Selecting the optimum specimen for assessing slight albuminuria, and a strategy for clinical investigation: novel uses of data on biological variation. Clin Chem.

[35] Gansevoort RT, Verhave JC, Hillege HL, Burgerhof JGM, Bakker SJL, de Zeeuw D (2005). The validity of screening based on spot morning urine samples to detect subjects with microalbuminuria in the general population. Kidney Int.

[36] Akerstrom M, Barregard L, Lundh T, Sallsten G (2014). Variability of urinary cadmium excretion in spot urine samples, first morning voids, and 24[thinsp]h urine in a healthy non-smoking population: Implications for study design. J Expos Sci Environ Epidemiol.

[37] Cirillo M, Laurenzi M, Mancini M, Zanchetti A, De Santo NG (2006). Low muscular mass and overestimation of microalbuminuria by urinary albumin/creatinine ratio. Hypertension.

[38] Mattix HJ, Hsu CY, Shaykevich S, Curhan G (2002). Use of the albumin/creatinine ratio to detect microalbuminuria: Implications of sex and race. J Am Soc Nephrol.

[39] Xu R, Zhang L, Zhang P, Wang F, Zuo L, Zhou Y (2008). Gender-specific reference value of urine albumin-creatinine ratio in healthy Chinese adults: Results of the Beijing CKD survey. Clin Chim Acta.

[40] Dyer AR, Greenland P, Elliott P, Daviglus ML, Claeys G, Kesteloot H (2004). Evaluation of measures of urinary albumin excretion in epidemiologic studies. Am J Epidemiol.

[41] Bakker AJ (1999). Detection of microalbuminuria. Receiver operating characteristic curve analysis favors albumin-to-creatinine ratio over albumin concentration. Diabetes Care.

[42] Lambers Heerspink HJ, Brantsma AH, De Zeeuw D, Bakker SJL, De Jong PE, Gansevoort RT (2008). Albuminuria assessed from first-morning-void urine samples versus 24-hour urine collections as a predictor of cardiovascular morbidity and mortality. Am J Epidemiol.

[43] Witte EC, Heerspink HJL, De Zeeuw D, Bakker SJL, De Jong PE, Gansevoort R (2009). First morning voids are more reliable than spot urine samples to assess microalbuminuria. J Am Soc Nephrol.

[44] Eshøj O, Feldt-Rasmussen B, Larsen ML, Mogensen EF (1987). Comparison of overnight, morning and 24-hour urine collections in the assessment of diabetic microalbuminuria. Diabet Med.

[45] Carrieri M, Trevisan A, Bartolucci GB (2001). Adjustment to concentration-dilution of spot urine samples: Correlation between specific gravity and creatinine. Int Arch Occup Environ Health.

[46] Elkins HB, Pagnotto LD, Smith HL (1974). Concentration adjustments in urinalysis. Am Ind Hyg Assoc J.

[47] Walser M (1987). Creatinine excretion as a measure of protein nutrition in adults of varying age. JPEN J Parenter Enteral Nutr.

[48] Jensen JS (1995). Intra-individual variation of overnight urinary albumin excretion in clinically healthy middle-aged individuals. Clin Chim Acta.

[49] Feldt-Rasmussen B, Mathiesen ER (1984). Variability of urinary albumin excretion in incipient diabetic nephropathy. Diabet Nephrop.

[50] Gomes MB, Gonçalves MFR (2001). Is there a physiological variability for albumin excretion rate?: Study in patients with diabetes type1 and non-diabetic individuals. Clin Chim Acta.

[CR51] The pre-publication history for this paper can be accessed here: http://www.biomedcentral.com/1471-2369/16/8/prepub

